# Global health diplomacy in humanitarian action

**DOI:** 10.1186/s13031-024-00605-5

**Published:** 2024-07-18

**Authors:** Luca Falqui, Fangfang Li, Yufeng Xue

**Affiliations:** International Committee of the Red Cross (ICRC) Regional Delegation for East Asia, 3-2 Qijiayuan Diplomatic Compound 9, Jianguomen Wai Dajie, Beijing, 100600 China

**Keywords:** Global health diplomacy, Humanitarian contexts, Universal health coverage

## Abstract

This commentary explores the intersection of Global Health Diplomacy (GHD) and humanitarian action within Fragility, Conflict, and Violence (FCV) contexts. It aims at addressing the multifaceted challenges faced by communities living in these environments, where a convergence of multiple factors, including over 110 active armed conflicts, creates complex emergencies impact on large populations globally. This commentary holds three primary significances: 1) it scrutinizes the profound and enduring health consequences of major humanitarian crises on last-mile populations, highlighting the pivotal role of health diplomacy for better navigating humanitarian challenges; 2) it advocates for a paradigm shift in humanitarian approaches, recognizing GHD’s potential in shaping international cooperation, building consensus on inclusive global health policies, and enabling more effective interventions; 3) it underscores the operational impact of health diplomacy, both at diplomatic tables and on the frontlines of humanitarian efforts. Through real-world cases such as the cholera outbreak in Yemen and the response to Ebola outbreaks in DRC, the paper illustrates how diplomatic dialogue can impact health outcomes in fragile settings.

Global Health Diplomacy (GHD) can play a significant role in enhancing humanitarian action. When the term 'humanitarian action' is used, it refers to the assistance and protection provided to save lives and alleviate the suffering of populations hit by natural disasters and/or living in situations of Fragility, Conflict and Violence (FCV) [1, 2]. When a context of FCV is compounded by factors such as poverty, food insecurity, climate shocks and public health emergencies, it can give rise to highly complex emergencies setting a ‘perfect storm’ [3, 4] of combined threats and vulnerabilities for the people affected.

According to the International Committee of the Red Cross (ICRC), there are approximately 110 active conflicts worldwide [5]. FCV situations nearly affect 1.9 billion people [2]. Many of them live in areas under the control of non-state actors [6] and can be hardly reached by national authorities and most humanitarian organizations [7].

In conflict settings, the impact of climate shocks is intensified, as these countries lack the necessary knowledge and financial resources to effectively cope with and adapt to the current climate-related crisis [8, 9]. Due to perceived difficulties and risks, many international institutions with the expertise and funding for implementing climate action continue to be extremely cautious when considering interventions in armed conflict contexts.

Furthermore, with an alarming 108 million individuals forcibly displaced in 2023 [10] and 365 million people currently requiring humanitarian aid for their survival [11], 200 million people every year could need humanitarian aid by 2050 [12], a doubling figure compared to 2018 and partly due to the climate crisis.

This bleak global picture has profound implications as it generates severe disparities in health outcomes and in the access to healthcare for many communities across the world. The persistence of barriers to essential health services represents one of the most significant challenges faced by global (public) health today.

## Addressing the health needs of last mile populations

Armed conflicts and other situations of fragility lead to high vulnerabilities [13, 14], particularly for communities in hard-to-reach areas and referred to as ‘last-mile’ populations. Beyond the immediate violent deaths and injuries caused by war and violence [15], the indirect health impact on these people is profound and enduring. The overall toll on human life is devastating [16, 17]. Moreover, when health workers, health facilities, or ambulances are not respected under International Humanitarian Law (IHL) and are targeted, the affected communities lose a last chance to access medical care [18].

The far-reaching political, economic, and humanitarian consequences of protracted armed conflicts raise serious concerns because livelihoods, infrastructures, provision of essential services, and economic activities are disrupted, and development is severely undermined.

The impact is higher when the resilience of these communities is overstretched by decades of hardships, such as in Yemen, Syria, Somalia, Afghanistan, Myanmar, or in the countries of the Great Lakes and the Sahel region.

When major public health emergencies occur, war-torn countries face growing challenges in addressing them. For the Democratic Republic of Congo (DRC), which has a long history of internal conflict, containing recurrent Ebola outbreaks has proven to be a critical undertaking. In areas controlled by non-state armed groups, access to essential healthcare services for civilians remains difficult, and widespread mistrust continues to exist towards aid coming from the central government or external sources.

An illustrative case is the Cholera outbreak occurred in Yemen in the period 2016-2022. This health crisis was primarily due to the disruption of the national health system caused by the long-standing internal conflict. The country was faced with the worst cholera epidemic of modern times, with more than 1 million suspected cases and 3000 deaths [19].

In Eastern DRC and Guinea, major obstacles in responding to Ebola outbreaks [20, 21] were observed when the local community showed resistance towards aid organizations and started to react with violence against community healthcare workers.

Amid the global emphasis on combating infectious diseases, a trend highlighted during the COVID-19 pandemic, particularly in fragile countries, was the persistent challenge to manage Non-Communicable Diseases (NCDs). In these contexts, local health systems rely on poor or inexistent NCD-related services. During the pandemic, the high prevalence of NCD in Low and Middle-Income Countries (LMICs) and in conflict settings [22, 23] resulted in severe discontinuity of care for diabetic, cancer, and cardio-vascular patients.

In the face of scarce life-saving assistance and protection, populations affected by armed conflicts not only face physical hardships, but also grapple with profound mental health and psycho-social consequences [24]. Pervasive uncertainty arising from forced and prolonged displacement has persistently posed challenges to these communities.

The separation from the loved ones, the absence of communication and connection with them, increase the profound psychological distress, trauma, and hardship [25], especially among children [26].

## The significance of (Global) health diplomacy

Global health confronts public health challenges that transcend national boundaries, with a notion of ‘health equity’ as its fundamental principle [27]. Unlike ‘equality’, which emphasizes on an equal level of healthcare for all, the strength of ‘equity’ lies in ensuring fair and just health outcomes by providing additional support to those less privileged and considered more vulnerable than other groups. The right to the highest attainable standard of physical and mental health is enshrined in several international legal instruments [28].

The International Health Regulations (IHR) and the future Pandemic Prevention, Preparedness and Response (PPPR) accord, emphasize this individual human right as a fundamental legal basis for the achievement of Universal Health Coverage (UHC).

As States negotiate revisions of the IHR and a new pandemics instrument, it is vital that the needs of people in humanitarian settings are considered. To that end, the ICRC has issued the following recommendations: a) explicitly include in the text populations affected by armed conflict and other humanitarian emergencies; b) explicitly mention International Humanitarian Law (IHL) as a relevant legal framework for these populations, as recognized by the WHO and other UN entities in recent resolutions and c) include provisions to address and prevent violence against health care in conflict situations and to protect the health workforce [29].

As we witness in Afghanistan, Sudan, Ukraine, Gaza and in many other contexts, the current plight of these populations reveals a stark reality: the aspiration for UHC and the achievement of the Sustainable Development Goals (SDG) agenda by 2030 remain highly elusive [30].

To realize global health goals, transdisciplinary solutions and effective international cooperation are not only desirable, but indispensable. This intention has been prominently evoked in global platforms, such as the World Health Summit in Berlin [31], the World Health Forum in Beijing [32], and highly resonated in recent deliberations of the UN General Assembly in New York or the World Health Assembly in Geneva.

Health equity remains a global challenge, both on the ground and in the application of existing international instruments.

The growing prominence of GHD over the years [33] however, shows that diplomatic negotiations are necessary to build consensus, strengthen international cooperation and agree on global health policies and interventions worldwide.

Achieving SDG #3 - Good Health and Well-being - requires grappling with multifaceted and complex issues. According to Kickbush et al., “GHD is a cross-disciplinary (national security, public health, international affairs, management, law, economics and trade policy), multilevel (government and non-government) and multistakeholder (civilians, community groups, non-governmental organizations) negotiation process that shapes and manages the global environment for health [34]”.

Effective health diplomacy exerted on influential global actors combined with active dialogue between developed and least developed nations, could increase the attainment of targets such as the UHC and help find agreements upon the Pandemic Treaty and the IHR. Strong health diplomatic mobilization becomes instrumental in keeping the promise of “leaving no one behind” and for improving the access to essential health services, particularly for the most vulnerable groups and the last mile populations.

## Health diplomacy for fragile countries

Health diplomacy extends well beyond the discussions among high-level diplomats and conferences. It is also manifested where humanitarian (health) workers negotiate in the field access to communities in need.

By operating in a two-way direction, GHD spans from the global sphere to local contexts and vice versa. In conflict zones and fragile environments, health diplomacy can become a source of motivation and guidance for frontliners and health professionals. Organizations such as the ICRC and Médecins Sans Frontières (MSF) have been playing a critical role in negotiating with local actors and arm bearers to gain humanitarian space, establish local partnerships and provide health services to hard-to-reach communities.

That represents health diplomacy in action, encompassing the navigation of complex security challenges, as well as agreeing with governments and non-state actors over health priorities and shared humanitarian goals.

When referred to humanitarian settings, health diplomacy intertwines with humanitarian diplomacy, adopting similar negotiating approaches and using public health analysis to meet the health needs of fragile and vulnerable populations. This dynamic interaction highlights the multifaceted nature of health diplomacy and emphasizes its operational impact both at the diplomatic tables and on the frontlines.

## A call for action

First, there is an urgent need for humanitarian aid organizations and global health institutions to invest in developing advanced communication and negotiation skills [35] within their workforce, particularly for the staff working in headquarters or holding operations management responsibilities.

The efficacy of these efforts hinges on their ability to communicate effectively with diverse stakeholders, to accommodate the demands of local communities and governmental authorities and agree upon policies and practices among a wide range of international organizations and donors. Advanced communication skills are critical to clarifying the relevance of global public health interventions, addressing possible concerns, mobilizing resources, and building trust between global and local actors.

Second, a regular exchange between the global and the local is vital. Health diplomats and public health experts in major capitals should consider cultural and contextual sensitivities, particularly in responding to worldwide humanitarian crises. Valuable insights stemming from community experiences can wield substantial influence in global deliberations and give voice to all stakeholders to improving UHC or agreeing upon pandemic treaties. This approach can also ensure the inclusiveness and transparency of global health policies. Without the knowledge from the field, the co-design and co-implementation of effective health interventions are impossible. In return, when informed policies transcend from the global to the field, and local ownership is sustained, improved health outcomes, stronger community resilience, and increased equity can be expected for the most underserved populations.

Third, it is crucial to acknowledge that the increasing number of armed conflicts is today compounded by factors such as underdevelopment, environmental degradation, economic insecurity, and public health crises. This combination characterizes most of the situations of Fragility-Conflict-Violence (FCV) observed at regional and global level. The consequent complex scenario demands additional resilience from the affected populations and calls for articulated and transdisciplinary responses from aid and development actors.

The increasing polarization and divide currently observed among global and middle powers raise high concerns about potential large-scale confrontations and underscores the urgent need for global solidarity and dialogue to prevent catastrophic humanitarian consequences.

In response to these concerns, the current debate focuses on ambitious Emergency Preparedness and Response (EPR) plans, on the delayed implementation of the 2030 agenda for SDG and the expectations towards proposals such as the Global Development Initiative (GDI) promoted by China [36] or the impact of future climate action and finance. Against this background, healthcare can transcend its role of essential service and become a vital connector and contributor to global development, human security, and peace [37].

By recognizing humanitarian health as an integral part of global health [38] and including health diplomacy into the broader domain of humanitarian diplomacy and policy, the international community has an excellent opportunity for capitalizing on evidence-based practices, inclusive policies, and international agreements to minimize more effectively the multiple and simultaneous global risks [39] affecting humanity in current times.

## Data Availability

Not applicable.
