# Correction: Global health diplomacy in humanitarian action

**DOI:** 10.1186/s13031-024-00609-1

**Published:** 2024-08-13

**Authors:** Luca Falqui, Fangfang Li, Yufeng Xue

**Affiliations:** International Committee of the Red Cross (ICRC) Regional Delegation for East Asia, 3-2 Qijiayuan Diplomatic Compound 9, Jianguomen Wai Dajie, Beijing, 100600 China

**Correction: Confict and Health (2024)18:46**

10.1186/s13031-024-00605-5

After the Abstract paragraph, the word “main text’ should be removed. All website links within the text needs to be removed and missing seven references have been updated.

States of Fragility 2020 | en | OECD [Internet]. www.oecd.org. Available from: https://www.oecd.org/dac/states-of-fragility-fa5a6770-en.htm.

^1^Pandemics and Armed Conflict: Recommendations for the Pandemic Accord [Internet]. International Committee of the Red Cross. 2023. Available from: https://www.icrc.org/en/publication/4732-pandemics-and-armed-conflict-recommendations-pandemic-accord.

^1^World Health Organization. Universal health coverage (UHC) [Internet]. World Health Organization. 2023. Available from: https://www.who.int/news-room/fact-sheets/detail/universal-health-coverage-(uhc).

^1^World Health Summit 2023 [Internet]. www.who.int. Available from: https://www.who.int/news-room/events/detail/2023/10/15/default-calendar/world-health-summit-2023.

^1^States of Fragility 2020 | en | OECD [Internet]. www.oecd.org. Available from: https://www.oecd.org/dac/states-of-fragility-fa5a6770-en.htm.

^1^stxu. ICRC engagement with armed groups in 2023 [Internet]. Humanitarian Law & Policy Blog. 2023. Available from: https://blogs.icrc.org/law-and-policy/2023/10/10/icrc-engagement-with-armed-groups-in-2023/.

Guidelines on Mental Health and Psychosocial Support [Internet]. International Committee of the Red Cross. 2017. Available from: https://www.icrc.org/en/publication/4311-guidelines-mental-health-and-psychosocial-support.

The original article has been corrected.

